# Chronic High Glyphosate Exposure Delays Individual Worker Bee (*Apis mellifera* L.) Development under Field Conditions

**DOI:** 10.3390/insects11100664

**Published:** 2020-09-27

**Authors:** Richard Odemer, Abdulrahim T. Alkassab, Gabriela Bischoff, Malte Frommberger, Anna Wernecke, Ina P. Wirtz, Jens Pistorius, Franziska Odemer

**Affiliations:** 1 Institute for Bee Protection, Julius Kühn-Institut (JKI), Federal Research Centre for Cultivated Plants, 38104 Braunschweig, Germany; abdulrahim.alkassab@julius-kuehn.de (A.T.A.); malte.frommberger@julius-kuehn.de (M.F.); anna.wernecke@julius-kuehn.de (A.W.); ina.wirtz@julius-kuehn.de (I.P.W.); jens.pistorius@julius-kuehn.de (J.P.); 2 Institute for Bee Protection, Julius Kühn-Institut (JKI), Federal Research Centre for Cultivated Plants, 14195 Berlin, Germany; gabriela.bischoff@julius-kuehn.de; 3 Odemer Apiaries, 73765 Neuhausen auf den Fildern, Germany; info@filderhonig.de

**Keywords:** honey bee health, glyphosate-based herbicides, brood development, field exposure, survival, overwintering, colony conditions, sublethal effects, residues

## Abstract

**Simple Summary:**

Glyphosate-based herbicides (GBH) can be found worldwide throughout conventional agroecosystems due to their unique and effective mode of action. Their use is generally not considered harmful to honey bees, and, consequently, foragers may encounter food sources that are potentially contaminated with GBH residues. However, recent studies found GBH to cause sublethal effects in bees, and therefore give rise to concern. While most related research has addressed such effects under laboratory conditions, field-realistic approaches under free-flying conditions are scarce. Here, we explore if GBH influences several important performance parameters at the colony level using standard and modified regulatory testing methods. Colony conditions (i.e., colony weight gain, individual worker bee survival, and overwintering) were not affected when subjected to chronic GBH exposure in a realistic range (high and low). In line with previous laboratory results, the high range of treatments revealed a delayed brood development of workers and reduced hatching weight of adults when compared with the control group. However, we concluded that more drastic effects on honey bee health did not seem to appear, as a broad range of performance parameters remained completely unaffected. In future research, the underlying mechanisms of the developmental delay that was confirmed here should be carefully investigated.

**Abstract:**

The ongoing debate about glyphosate-based herbicides (GBH) and their implications for beneficial arthropods gives rise to controversy. This research was carried out to cover possible sublethal GBH effects on the brood and colony development, adult survival, and overwintering success of honey bees (*Apis mellifera* L.) under field conditions. Residues in bee relevant matrices, such as nectar, pollen, and plants, were additionally measured. To address these questions, we adopted four independent study approaches. For brood effects and survival, we orally exposed mini-hives housed in the “Kieler mating-nuc” system to sublethal concentrations of 4.8 mg glyphosate/kg (T1, low) and 137.6 mg glyphosate/kg (T2, high) over a period of one brood cycle (21 days). Brood development and colony conditions were assessed after a modified OECD method (No. 75). For adult survival, we weighed and labeled freshly emerged workers from control and exposed colonies and introduced them into non-contaminated mini-hives to monitor their life span for 25 consecutive days. The results from these experiments showed a trivial effect of GBH on colony conditions and the survival of individual workers, even though the hatching weight was reduced in T2. The brood termination rate (BTR) in the T2 treatment, however, was more than doubled (49.84%) when compared to the control (22.11%) or T1 (20.69%). This was surprising as T2 colonies gained similar weight and similar numbers of bees per colony compared to the control, indicating an equal performance. Obviously, the brood development in T2 was not “terminated” as expected by the OECD method terminology, but rather “slowed down” for an unknown period of time. In light of these findings, we suggest that chronic high GBH exposure is capable of significantly delaying worker brood development, while no further detrimental effects seem to appear at the colony level. Against this background, we discuss additional results and possible consequences of GBH for honey bee health.

## 1. Introduction

Glyphosate-based herbicides (GBH) were introduced to the market almost fifty years ago, with a unique mode of action that is superior to most other active ingredients [1]. Its high efficiency and the easy-to-apply practice have led to the increasing use of GBH during the past three decades. In numbers, this means that over 820 million kilograms of glyphosate were globally applied in 2014, where the last decade alone accounted for 6.1 billion kilograms (reviewed in [2,3]). Predominantly used on farmland, the agricultural utilization of GBH includes, but is not limited to, pre-sowing, pre-harvest (e.g., desiccation), and stubble application. It reduces labor and machine costs, whereas other pesticides intend to improve crop yields. Accordingly, GBH is far more than just a weed killer and must be seen as an agronomical tool implemented in the working practice of farmers worldwide [4].

Recently, German beekeepers have become concerned about residues of the herbicide in their honey not only putting its marketability at risk but also leaving open the question of adverse effects on bee health. In the state of Brandenburg, a harvest of 4000 kg honey was found to be substantially contaminated with 76 mg glyphosate/kg in 2020 [5]. This honey had to be withdrawn from the market, as the maximum residue level (MRL) of glyphosate is limited to 0.05 mg/kg in the European Union [6]. In Canada, Thompson et al. [7] found the herbicide in 197 of 200 samples of honey that they examined, with a maximum concentration slightly below the European MRL. To date, studies reporting field-relevant GBH residues in bee products are scarce, and, so far, this particular case in Canada was higher than what was reported in the limited number of studies done over the last five years in other countries [8,9,10]. By reason of the abovementioned utilization, GBH is usually not applied during flowering, and yet field crops represent a potential source for honey bee exposure. Due to the control of weeds within or adjacent to these fields, pollinators can become unintentional targets of control measures, transferring back residues to their nests. Grassland conversion and spray drift could also be a possible route of exposure [11], so are domestic and non-agricultural use, including weed control in rail traffic, parks, and home gardens [12].

For many years, it was believed that GBH bore no risk for honey bees because glyphosate’s target enzyme 5-enolpyruvylshikimate 3-phosphate synthase (EPSPS) was not found in insects or other animals [13]. In accordance with results from a regulatory risk assessment, a hazard for honey bees could not be identified, and GBH were therefore classified as “not harmful to bees” in Germany. The oral and contact LD_50_ for glyphosate are determined to be 100 and >100 µg/bee, indeed suggesting low acute toxicity [14]. More recently, however, several laboratory studies reported sublethal effects of the herbicide at the individual bee level. Such effects are of current concern, as they can reduce honey bee reproduction, immunity, cognition, and overall physiological functioning, leading to a suboptimal honey bee performance and population decline [15]. For GBH, impaired cognitive abilities and navigation skills [16,17], and, primarily, implications for the host-microbiome were indicated [18,19,20]. While microbiomes’ consequences for bee health are still under discussion, affected physiological parameters are more worrying. In worker bees, GBH seem not only to hypertrophy and damage royal jelly-producing glands [21] but also to delay molting and reduce larval weight, suggesting serious adverse effects at the colony level [19,22].

However, these findings were obtained away from a realistic in-hive environment and would not necessarily translate to the colony level [23]. In eusocial insects, past studies have shown that the fate of individuals was uncoupled from the fate of their colony [24]. Social buffering, i.e., tolerating the loss of individuals as long as the functionality and reproduction of the colony are maintained, is key for the superorganisms’ susceptibility towards stressors, including pesticide exposure [25]. Therefore, only limited insight can be gained by confronting individuals, but not the whole colony, to such stressors. Yet, the only study investigating GBH exposure under field conditions was presented by Thompson et al. [26]. Free-flying colonies were fed a sucrose solution spiked with technical-grade glyphosate, according to a realistic range. Brood development was assessed until day 16 without evidence of detrimental effects, either on the brood and adult mortality or on the pupal weight. However, a full brood cycle (21 d) or adult survival after chronic exposure was not yet covered. Moreover, the results obtained with technical-grade glyphosate cannot be equated with exposure to the formulated product. Past studies investigating the effects of spray adjuvants showed that these “inert” coformulations may have a more severe effect on bee health than previously assumed [27,28]. This means that the formulated product should be preferred to the active ingredient when studying GBH effects. To date, most standard residue analyses do not include screening for glyphosate. One of the first methods to be described was published quite recently [10] and is not yet integrated with any sort of multi-method suitable for quick screening purposes. We currently see residues ranging from 0.02 to 120 mg/kg in honey but cannot say how pervasive GBH are in other bee-relevant matrices [8,9,10], partly because test methods have been lacking. In light of these global findings, we assume that colonies are not only exposed acutely but rather chronically, to GBH. Brood may be raised with contaminated food and colonies overwinter on contaminated stores as a consequence. Hence, the impact of GBH on colony performance parameters such as brood development or overwintering success under chronic conditions still needs to be answered and may play a key role in the risk assessment of formulated glyphosate products.

This study seeks to obtain data, which will help to address these research gaps. With four independent study approaches, we thus examined combined individual and colony-level parameters under field conditions. Based on previous laboratory studies indicating sublethal effects, we hypothesized that (i) larval weight loss should be translated to adult workers after chronic exposure of the colony, and, (ii) if overwintering and/or survival are affected, a clear negative impact on colony conditions and mortality in individually followed workers should become visible. Furthermore, delayed molting (iii) must be reflected by brood development, either with a reduced amount of brood or even a higher rate of terminated brood cells. According to these hypotheses, mini-hives were challenged to a low and high concentration of GBH-spiked food in our key experiment “brood development and survival”. Two supporting experiments, “field exposure” and “overwintering”, were carried out using modified regulatory risk assessment methods [29,30,31], in order to measure the population dynamics of GBH-exposed full-sized colonies. In addition, we expected to confirm/expand (iv) field-realistic GBH residue data in different bee matrices to complete the picture of a realistic exposure scenario. Employing a semi-field and field approach, we aimed to collect residues of glyphosate and its metabolite aminomethylphosphonic acid (AMPA) in a realistic foliar spray setting.

## 2. Experiment 1: *Brood Development and Survival*

### 2.1. Methods

#### 2.1.1. Experimental Colonies and Field Site

**Figure 1:**
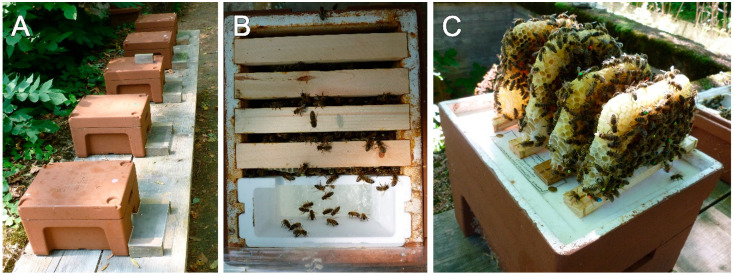
(**A**) Mini-hives of one treatment group including five colonies during GBH exposure. (**B**) Plan view of a mini-hive with four top-bars and a feeding device, providing space for an intact honey bee colony. (**C**) For the brood/survival assessments, all four combs were removed, and both sides of each top-bar were photographed without/with bees attached, respectively (see Section 2.1.4 and Section 2.1.5).

This field study was conducted from June to September. We used the “Kieler mating-nuc” system, a Styrofoam box with four top-bars, a strip of a beeswax foundation attached to it, and a feeder equipped to it (Figure 1A–C), recently presented in Odemer et al. [32]. In brief, 19 mini-hives were established, with about 800 worker bees originating from the brood frames of two healthy donor colonies with no clinical symptoms of adult bee or brood diseases visible during inspection. Subsequently, unmated sister queens (*Apis mellifera* L.) were introduced, and the mini-hives were placed at a remote apiary near Wurmberg, Germany for mating after confinement in a dark and chilled room for 24 h. The corresponding coordinates were as follows: latitude 48.867414°, longitude 8.808538°. Within proximity of 250 m, no other hives were set up, whereas in an extended radius (>250 m) a sufficient number of unrelated colonies were located to provide enough drones for mating. At the present time, bees could forage on *Tilia* spp., *Cyanus segetum,* and other floral sources in the surroundings, as the weather provided favorable conditions (avg. outdoor temperature 18.9 °C, precipitation 53.97 L/m², [33]).

Within five weeks, the established hives showed all the relevant brood stages (eggs, larvae, and patches of sealed brood) and newly built combs as a sign of successful mating and colony establishment. In total, fifteen of the mini-hives were randomly assigned to the control and the two treatments T1 and T2, five (= replicates) to each group, respectively. One T2 mini-hive was discovered to be queenless shortly after the start of the experiment and was therefore removed (n: C = 5, T1 = 5, T2 = 4). We maintained the remaining four mini-hives from a total of 19 without further treatment. They served as receiver colonies for the subsequent survival assessment of the experiment (see Section 2.1.5).

#### 2.1.2. Chemical Treatment and Sampling

Glyfos Unkraut-Frei^®^ (Dr. Stähler, Köln, Germany) with a glyphosate concentration of 360 g a.i./L was used for the treatment. Accordingly, the product was directly mixed into 5 L of feeding syrup (Apiinvert, Südzucker GmbH, Mannheim, Germany) to achieve the desired concentrations. The treatment was comprised of two feeding regimes derived by residue data reported in nectar and honey [5,7,8,9,10]. We wanted to cover the range of 5 to 150 mg/kg in order to represent field-realistic exposure, resulting in groups T1 having a measured concentration of 4.8 mg a.i./kg and T2 having a measured concentration of 137.6 mg a.i./kg feeding syrup. This equaled 0.090 g product/5 L for T1 and 2.610 g product/5 L for T2. The control was fed with untreated syrup, which was provided in a weekly interval like in all other groups. Chronic treatment in T1 and T2 was maintained for 21 consecutive days to cover a full worker-brood cycle with a total amount of 0.81 kg feeding syrup per hive. This corresponded to a calculated amount of T1: 3.859 mg and T2: 111.428 mg glyphosate per hive, respectively.

Before the experiment, a sample of each feeding regime and the untreated feeding syrup was collected, respectively. On day 21, pooled samples of the stored syrup from all groups were collected from in-hive storage cells for residue analyses.

#### 2.1.3. Colony Conditions

The total number of bees was estimated for each colony according to the “Liebefeld method” [34]. In addition, the absolute hive weight was recorded at the time of the colony assessments with a precision scale (Mettler-Toledo Kompaktwaage ICS445k, Albstadt, Germany, resolution 0.01 g). The assessments were performed shortly before the first application on 31 July (Day after Application = DAA0), 30 August (DAA30), and 5 September (DAA56).

#### 2.1.4. Brood Development and Photographic Assessment

For the colony condition assessment before the first application of the test item (DAA0), one or two brood combs were taken out of each replicate from the control, T1, and T2 (Brood area Fixing Day = BFD) groups, with a sufficient amount of the desired brood stage. The development of the bee brood was continuously assessed and followed by selecting approximately 100 cells per replicate containing eggs. Pictures were taken from the comb-area containing eggs and were modified according to Schur et al. [31]. In brief, selected combs were uniquely identified, and the fixed brood area was photographed during each brood stage assessment (BFD assessments, Canon EOS 700D with Canon EF-S 24 mm F2.8 pancake lens). This way, each selected cell was identified to evaluate its content in the digital images in order to follow the development process. Cells were classified and evaluated following the scheme in Table S1. From this evaluation, the brood termination rate (BTR—for details see Supplementary Material S1) was respectively calculated. The original method was further modified, so that only three assessments were necessary in order to reduce invasive operations and the disturbance of the colonies.

#### 2.1.5. Survival of Individual Bees and Hatching Weight

After the 21 d chronic exposure, one sealed brood comb containing brood cells ready to hatch was removed from the mini-hives of all groups and put together treatment-wise—five combs from control and T1, and four from T2—in an incubator for 24 h (Memmert IN30, Schwabach, Germany at 33 °C, 70% RH, total darkness). Subsequently, all hatched bees were pooled treatment-wise, and 30–40 young workers were collected at random. Bees’ hatching weight was documented at random with a portable precision scale (Kern CM 60-2N, Balingen, Germany, resolution 0.01 g). Therefore, single bees were transferred carefully into a tared container. They were weighed according to their treatment and respective pool, resulting in a total number (n) of control (50), T1 (38), and T2 (36).

For identification, a colored and numbered opalith plate was glued on the thorax using shellac. In addition, we marked the dorsal side of the abdomen with a hive-specific color (Posca Marking Pen, Japan) in order to identify drifting bees that entered neighboring colonies. The bees were then introduced into four of the mini-hives (=replicates) remaining from the initial 19 and were divided equally according to their treatment. The total number (n) of introduced individuals per treatment was as follows: control (152), T1 (149), and T2 (141). Then, survival was monitored for a period of 25 days. See Supplementary Figure S2 for a detailed experimental setup scheme. The monitoring started 24 h after the bees were introduced. The mortality assessment included a check every two to three days, for which all combs, including the inside of the hive, were photographed for the subsequent counting of the marked bees on a computer screen. The pictures were taken outside the foraging activity, early in the morning.

#### 2.1.6. Statistical Analyses

The estimated number of bees and brood cells from all the colony condition assessments, the hatching weight and weight gain of the mini-hives as well as the brood termination rate, and the number of selected eggs from the BFD were checked with a Shapiro–Wilk test for normal distribution. If the data were normally distributed, a one-way ANOVA was performed to compare multiple experimental groups, respectively. Statistically significant results were further tested pairwise with a Student’s *t*-test. This test was also used when only two normally distributed groups required comparison. If the data were non-normally distributed, a Kruskal–Wallis test was performed to compare multiple experimental groups, respectively. Statistically significant results were further tested pairwise with a Wilcoxon rank-sum test. This test was also used when only two non-normally distributed groups required comparison. To correct for multiple comparisons, *p*-values were adjusted with the Bonferroni method.

Mortality in the mini-hives was evaluated with a Kaplan–Meier survival analysis (KM). Survivorship between control and treatments was compared pairwise and tested for significance with log-rank tests (Cox–Mantel) using the “pairwise_survdiff” function [35]. Individuals remaining alive at the end of the experiment were considered censored, as were those observed but not collected on the final day. All groups that underwent the KM analysis, including the four replicates used in the monitoring phase, were additionally compared using a Cox proportional hazards model to determine the hazard ratio (HR). Possible inter colony effects were evaluated as a covariate to justify data pooling of the same treatments.

All analyses were performed in R v.3.6.2 [36] with the packages *survival* v.2.3.8 [35], *survminer* v.0.4.6 [37], and *ggpubr* v.0.2.4 [38]. A significance level of α = 0.05 was used for all tests, respectively.

### 2.2. Results

#### 2.2.1. Colony Conditions

**Figure 2:**
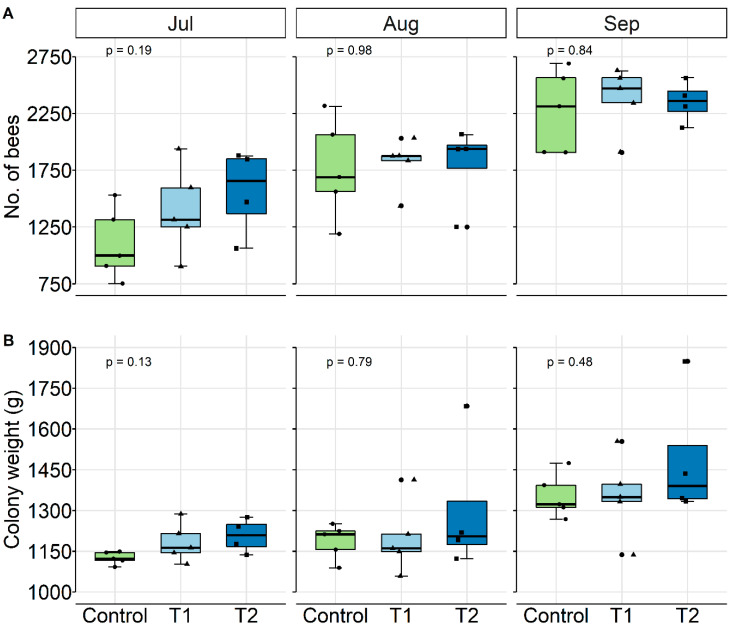
Effect of chronic GBH feeding exposure (T1: 4.8, T2: 137.6 mg a.i./kg) on colony conditions, displayed as a boxplot. (**A**) Number of worker bees increased similarly and was not significantly affected by GBH (*p* > 0.05, ANOVA)**.** (**B**) Absolute colony weight and its increase during the study. There were no significant differences between groups (*p* > 0.05, Kruskal–Wallis test).

The complete randomized assignment of the mini-hives to their respective treatment caused the initial colony strength to differ between groups, though not significantly, with the control being the weakest (Figure 2A, Jul, *p >* 0.05, ANOVA). At the time of the following two assessments, the control group was able to compensate this deficit by establishing equivalent numbers of bees, similar to the other groups (Figure 2A, Aug, Sep). With the progression of the study, the absolute colony weight was similarly increasing, with no significant differences between groups (Figure 2B, *p >* 0.05, Kruskal–Wallis test).

#### 2.2.2. Brood Development and Photographic Assessment

With reference to the developing time of a worker honey bee from egg to adult (21 to ±1 days [39,40]), it was assumed that all eggs should completely be developed at the time of the last assessment date (BFD+21). The cumulative number of selected eggs from all replicates was control (n = 522), T1 (n = 525), and T2 (n = 426), with no significant difference of the mean distribution amongst the control (n = 104/replicate), T1 (n = 105/replicate), and T2 (n = 106/replicate) groups (Supplementary Figure S3, *p* > 0.05, ANOVA).

**Figure 3:**
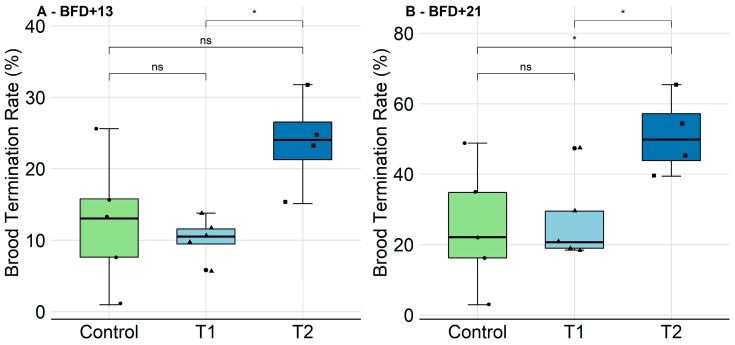
Effect of chronic GBH feeding exposure (T1: 4.8, T2: 137.6 mg a.i./kg) on the Brood Termination Rate (BTR; see Supplementary Material Method S1), displayed as a boxplot. (**A**) The BTR of treatments T1 and T2 did not show significant differences when compared to the control (*p* > 0.05, *t*-test, pairwise) on BFD+13 (Brood area Fixing Day). (**B**) Eight days later, on BFD+21, T1 did not show differences when compared to the control (*p* > 0.05, *t*-test, pairwise). T2, however, revealed a significantly higher BTR (*p* < 0.05, *t*-test, pairwise).

In the control, T1, and T2 groups, successful development was observed in the majority of the marked brood cells. On BFD+13, the median termination rate was 13.04/10.53% in the control and T1, respectively. In the test item treatment T2, brood termination was not significantly increased at this point when compared to the control (24.05%, Figure 3A).

On BFD+21, the median termination rate was 22.11/20.69% in the control and T1, respectively. In the test item treatment T2, brood termination was significantly increased when compared to the control (*p* < 0.05, *t*-test, pairwise). The median termination rate was 49.84% (Figure 3B). The full detail of the brood assessments is provided with the Supplementary Materials (Figure S4 and Method S1).

#### 2.2.3. Survival of Individual Bees and Hatching Weight

**Figure 4:**
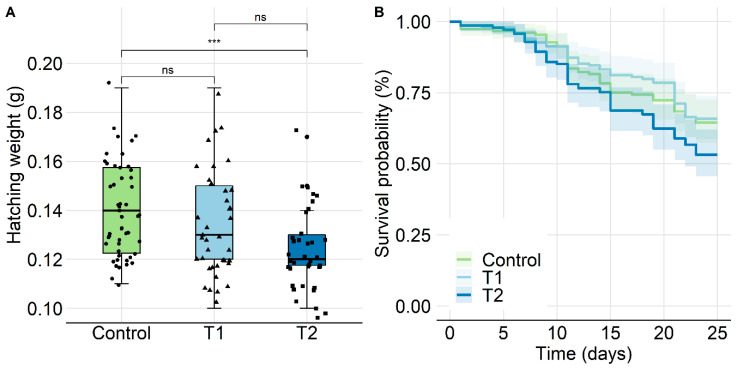
Effect of chronic GBH feeding exposure (T1: 4.8, T2: 137.6 mg a.i./kg) on hatching weight and survival of adult worker bees. (**A**) Hatching weight was assessed on the day of emergence after 24 h in the incubator and displayed as boxplot. Weight in T2 was significantly lower (16.7%) when compared to the control (*p* < 0.01, Wilcoxon rank-sum test, pairwise). (**B**) Survival of individual workers is illustrated with a Kaplan–Meier survival curve and 95% confidence intervals. A pairwise comparison with corrections for multiple testing did not confirm differences between the respective treatments and the control (*p* > 0.05, log-rank test, pairwise).

The hatching weight of adult workers in T2 was significantly lower when compared to the control (*p* < 0.01, Wilcoxon rank-sum test, pairwise) with median values of control: 0.14, T1: 0.13, and T2: 0.12 g. Therefore, T2 revealed a reduction in emergence mass when compared to the control and T1 by 16.7 and 8.3%, respectively (Figure 4A). Furthermore, a Kaplan–Meier-Survival analysis employing pairwise comparisons between group levels with corrections for multiple testing showed no significant differences between the respective treatments and the control (Figure 4B, *p* > 0.05, log-rank test, pairwise). In addition, a Cox proportional hazards model was applied to determine the hazard ratio (HR), displayed as a forest plot (Supplementary Figure S5). With an HR of 0.93 for T1 and 1.43 for T2, the treated bees were not expressing higher mortality when compared to the control (T1: *p* = 0.73, T2: *p* = 0.051, log-rank test). To justify pooling bees from the same groups but different mini-hives for the survival analysis, these hives were evaluated separately and were treated as replicates. The test showed no significant differences (Supplementary Figure S6, *p* > 0.05, log-rank test).

## 3. Experiment 2: *Field Exposure*

### 3.1. Methods

#### 3.1.1. Experimental Colonies and Field Sites

This field study was conducted from July to August. Twelve honey bee colonies (*A. mellifera*), healthy and queen-right with one hive body including ten combs were used. The colonies were as homogeneous as possible at a strength of approximately 15,000 bees per colony. Queens originated from one breeding line (sisters, reared at the test facility in the same year). No clinical symptoms of adult bee or brood diseases were visible during inspection. Colonies were split into two groups with six replicates each (control and treatment T).

Eight days before application (Day After Treatment = DAT-8), the colonies were placed at the edge of two flowering *Phacelia tanacetifolia* plots near the city of Braunschweig with an area of approximately 1 ha, respectively. The corresponding coordinates were as follows: Control plot C; latitude 52.296415°, longitude 10.437062°, Treatment plot T; latitude 52.202098°, longitude 10.623331° (beeline from field to field: 16.45 km). Colonies from the treatment plot were migrated after nine days of exposure (DAT+8) to the control plot C.

#### 3.1.2. Chemical Treatment and Sampling

Roundup^®^ Power Flex (Monsanto Agrar Deutschland GmbH, Düsseldorf, Germany) with a glyphosate concentration of 480 g a.i./L was used for the treatment. The treatment was applied with a rate of 3.75 L/ha in 300 L water/ha on the flowering phacelia (BBCH 64–65) plot T (DAT0, 26 July) with a field sprayer. The control remained unsprayed.

A total of 12 beebread (stored pollen), 60 food (stored syrup), and 10 plant samples (whole plant except the roots) were taken at different time intervals before and after the treatment from both plot sites for residue analyses.

#### 3.1.3. Colony Conditions

The colony conditions were assessed on three dates using the “Liebefeld method” [23]. On DAT-6 (20 July) before the exposure and DAT+15 (10 August) and DAT+57 (21 September) after the exposure, respectively. The time intervals were chosen to cover colony conditions before and after the treatment and to include more than one brood cycle. Parameters such as the number of bees, brood cells, and stores (honey and pollen) were estimated according to the method.

#### 3.1.4. Statistical Analyses

Please see Section 2.1.6.

### 3.2. Results

#### Colony Conditions

**Figure 5:**
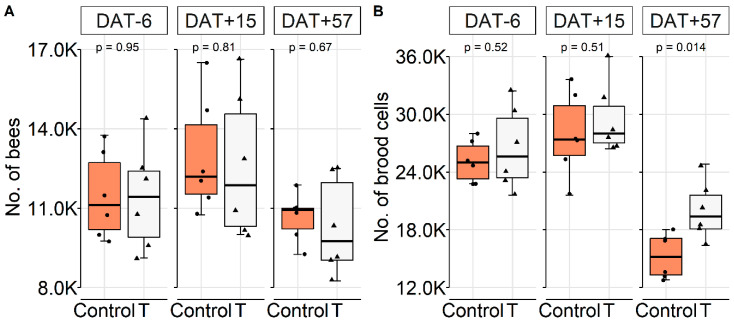
Effect of GBH field exposure (foliar spray) on colony conditions, displayed as boxplot. (**A**) Number of worker bees. (**B**) Number of brood cells during the study. On DAT+57, the treatment T had significantly more brood cells when compared to the control (Wilcoxon rank-sum test). For all other assessments, conditions were not significantly different. Dates correspond to the following months: DAT−6 (=20 July), DAT+15 (=10 August), and DAT+57 (=21 September).

Overall, colonies were well provided and did not suffer food shortage (Supplementary Figure S7). GBH did not significantly affect colony development in this experiment, regardless of the type of exposure and time of the year (*p* > 0.05, *t*-test). Bees and brood showed a pattern of increases and decreases in an alternate sequence (Figure 5A,B). However, brood cells were decreasing faster in the control on DAT+57 due to a greater winter food intake (Figure 5B, Supplementary Figure S7, *p* = 0.014, Wilcoxon rank-sum test).

## 4. Experiment 3: *Overwintering*

### 4.1. Methods

#### 4.1.1. Experimental Colonies and Field Sites

This field study was conducted from October until March of the following year. The same setup as in Section 3.1.1 was applied, with one exception: Hives were at a strength of approximately 10,000 bees per colony. All colonies were located at a remote apiary near the city of Braunschweig. The corresponding coordinates were as follows: latitude 52.202098°, longitude 10.623331°.

#### 4.1.2. Chemical Treatment and Sampling

Roundup^®^ Power Flex (Monsanto Agrar Deutschland GmbH, Düsseldorf, Germany) with a glyphosate concentration of 480 g a.i./L was used for the treatment. The treatment was comprised of one feeding regime: T with a nominal concentration of 8.13 mg a.i./kg (according to Motta et al. [18]). Accordingly, the product was directly mixed into 5 L of the feeding syrup (1:1 sugar water, *w*/*w*, density: 1.2296) for each colony to achieve the desired concentration, respectively. The control was fed with untreated syrup. Chronic treatment in T was maintained until the feeder was emptied. The average measured concentration of 5.439 mg a.i./kg in the feeding solution corresponded to a calculated total amount of T: 33.436 mg glyphosate per hive.

A total of 66 samples of stored food were collected from in-hive storage cells from all groups for residue analyses at different time intervals.

#### 4.1.3. Colony Conditions

The colony conditions were assessed on three dates using the “Liebefeld method” [34]. On DAT-1 (1 October) before the exposure and DAT+43 (14 November) and DAT+170 (21 March) after the exposure, respectively. The time intervals were chosen to cover colony conditions before and after the treatment, with a focus on during and after overwintering. Parameters such as number of bees, number of brood cells, and the number of stores were estimated according to the method.

#### 4.1.4. Statistical Analyses

Please see Section 2.1.6.

### 4.2. Results

#### Colony Conditions

**Figure 6:**
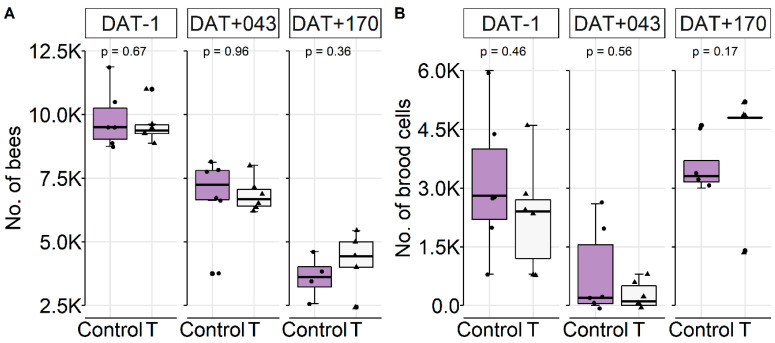
Effect of GBH exposure (feeding) on colony conditions over winter, displayed as boxplot. (**A**) Number of worker bees. (**B**) Number of brood cells during the course of the study. For all assessments, conditions were not significantly different. Dates correspond to the following months: DAT−1 (=1 October), DAT+43 (=14 November), and DAT+170 (=21 March).

Shortly before hibernation (DAT+43), brood activity was reduced in all colonies independent of their treatment (Figure 6B), followed by a continuous decrease of bees (Figure 6A). At the end of overwintering, a decrease of stored food was observed (Supplementary Figure S8) as a result of the returning brood activity (Figure 6B). GBH did not significantly affect colony development in this experiment, regardless of the type of exposure and time of the year (*p* > 0.05, *t*-test). On DAT+170, we found that two colonies in the control and one in T had not survived winter.

## 5. Experiment 4: *Determination of GBH Residues*

### 5.1. Methods

#### 5.1.1. Experimental Colonies and Field Sites

This semi-field study was conducted from July to August. The same setup as in Section 3.1.1 was applied, with the following exceptions: Queens were removed on DAT-14 to stimulate foraging in the tunnel tents. Colonies were split into two groups, with three replicates each (control and treatment T).

On the day of application (DAT0, 2 August), each colony was placed in a tunnel tent with an area of approximately 33.5 m² on a flowering *P. tanacetifolia* plot at our field site in Braunschweig, respectively. The corresponding coordinates were as follows: latitude 52.296415°, longitude 10.437062°. Due to the loss of forage in the GBH-treated tents, all colonies were migrated after 13 days of exposure (DAT+13, 15 August) to a remote apiary.

#### 5.1.2. Chemical Treatment and Sampling

Roundup^®^ Power Flex (Monsanto Agrar Deutschland GmbH, Düsseldorf, Germany) with a glyphosate concentration of 480 g a.i./L was used for the treatment. The treatment in the T tents was applied with a rate of 3.75 L/ha in 300 L water/ha (DAT0) on the flowering phacelia (BBCH 65) using a portable boom sprayer (Schachtner, München, Germany). The control was sprayed with water.

A total of four stored food, two pooled honey sac, two pooled corbicular pollen, and four pooled plant samples were taken at different time intervals after the treatment from all tents for residue analyses. On DAT+5, plants could no longer be sampled due to the action of the herbicide.

#### 5.1.3. Residue Analyses

The samples of all the experiments (1–4) were analyzed together using a method without a derivatization step based on the methods for honey, milk, soybeans, and maize published by Chamkasem et al. [41,42,43,44]. Depending on the sample material, some modifications were necessary. The method was validated by analyzing a series of spiked replicate honey, pollen, and plants (phacelia) samples (see Supplementary Materials S2).

LC-MS/MS was used to identify and quantify glyphosate (GLY) and its metabolite aminomethylphosphonic acid (AMPA) in the samples. The system that was used was a Nexera X2 HPLC system (SHIMADZU Corp., Kyoto, Japan) coupled to a triple quadrupole mass spectrometer Q TRAP 6500+ (SCIEX, Framingham, MA, USA) equipped with an electrospray ionization (ESI) source. The mass spectrometer was operated in the negative ESI mode, and three multiple reaction monitoring (MRM) transitions were monitored for each analyte in order to confirm the compound identity. In undiluted and 1:10 diluted samples, the analyte contents were determined using matrix-matched standards. If samples had to be diluted 1:100 or 1:1000, the analytes were quantified using reference standards in an extracting agent, as the matrix effects were sufficiently reduced by dilution. For quantification, the internal standard method was used, with Glyphosate-^13^C2^15^N and AMPA^13^C^15^N as the internal standards [45]. This procedure minimizes the matrix effect and enables accurate quantification.

The average recoveries in plants and pollen at the fortification levels of 25, 50, and 250 µg/kg were between 73% and 83% for GLY and 71% and 87% for AMPA, with relative standard deviations (RSD) of less than 20% for both analytes. An exception was the recovery of 148% (RSD 18%) for GLY in pollen at the limit of quantification of 25 µg/kg. For GLY and AMPA, the average recoveries in honey at the fortification levels of 25 µg/kg, 250 µg/kg, and 50 mg/kg were in the range of 74% and 112%, with RSDs between 9% and 17%.

In honey and plants, the LOD of GLY was 5.0 µg/kg and in pollen, it was 12.5 µg/kg, respectively. The LOQ was 12.5 µg/kg and 25 µg/kg, respectively. In honey, the LOD of AMPA was 2.5 µg/kg, and in plants and pollen, it was 12.5 µg/kg, respectively. The LOQ was 5.0 µg/kg and 25 µg/kg, respectively. For details of the analytical method and the method validation, see Method S2 in the Supplementary Materials.

### 5.2. Results

The results of the residue analyses from all experiments (1–4) for glyphosate and its metabolite aminomethylphosphonic acid (AMPA) are presented as an overview in Table 1. A total of 176 samples were measured, including controls (experiment 1: six samples, experiment 2: 92 samples, experiment 3: 66 samples, experiment 4: 12 samples) (see raw data [46]). It could be confirmed that all experimental colonies from the respective treatments were exposed to glyphosate, respectively.

In experiments 1, 2, and 3, irrespective of the time interval after the application (short term: three days, or long term: 170 days), glyphosate residues remained constant in all the sugar matrices. In experiment 2, a 15.9-fold average increase, and a 24-fold peak increase (see raw data [46]) was measured in stored pollen (beebread) when compared to stored nectar. In plants, a range similar to experiment 4 (tunnel tents) was measured shortly after the application on DAT0. Degradation of glyphosate in the tunnel tents, however, was faster than in the field. In experiment 4, a matrix-dependent exposure gradient could be identified, which could be presented from high to low glyphosate residues, as follows: corbicular pollen > plants > honey sac > stored food/nectar. Honey sac residues were measured with a 3.3-fold reduction when compared to their floral source (plants). In turn, a 27.4-fold increase was measured in pollen when compared to honey sac contents, similarly to what was reported in experiment 2 for the stored products.

Notably, in experiment 1, trace residues of glyphosate were found in the pooled control food on DAT+21 (0.18 mg a.i./kg, AMPA was not detectable). Furthermore, in experiment 3, trace residues of glyphosate were found in one control and one treatment colony before the application on DAT−1 (C2: 0.05 and T2: 0.04 mg a.i./kg, AMPA was not detectable).

## 6. Discussion

Our study provides valuable information about the chronic effects of GBH exposure on important performance parameters of honey bees (*A. mellifera*) under real colony conditions. The chronic oral exposure of mini-hives to a high concentration (T2, 137.6 mg a.i./kg) of the formulated product had subtle but indirectly detectable effects on brood development. Whereas the survival of individuals under such conditions was not affected, exposure in T2 also resulted in a significant reduction of the hatching weight in worker bees.

Overall, the colonies of our different study approaches did not collapse during winter, nor were any other conditions (i.e., weight gain, bee and brood production) affected when directly foraging on GBH-sprayed crops or consuming GBH-spiked food. At least not with the formulated products that were used here. Colony development followed a typical pattern considered normal for a temperate climate in all experiments [47]. To our surprise, the brood termination rate (BTR) was significantly increased on BFD+21 after the brood was chronically exposed to the high concentration (T2) in the feeding phase of experiment 1. A trend became evident eight days earlier at BFD+13, suggesting that the duration of exposure may have played a crucial role in this effect. It is particularly interesting that the number of bees and the colony weight did not decline but increased during the study, despite the almost doubled BTR in T2. Our finding is supported by Vazquez et al. [22], who reported similar effects when larvae were exposed to chronic GBH feeding *in vitro*, as they stated that molting was delayed and that the fresh weight was reduced. Similarly, the hatching weight of young bees in our study was significantly reduced in the high concentration (T2). With a median of 120 mg, however, the weight remained within the variation of reported values in the literature; Kunert and Crailsheim [48], for instance, found the mean fresh weight of summer bees to be 116.7 mg (±1.5 SE), where Żółtowska et al. [49] stated a range from 86.08 mg to 121.18 mg. In contrast, Thompson et al. [26] found larvae/pupae not to be affected by GBH, nor was there a negative impact on the pupal weight when using the feeding method described by Oomen et al. [29]. Therefore, the authors decided to only conduct brood assessments until day 16 of development in their study, which does not fully cover a complete worker brood cycle. Moreover, it needs to be emphasized that for their brood study, technical grade isopropylamine (IPA) salt was used, instead of a formulated glyphosate product. This can be of relevance in relation to the differences in the outcome of Vazquez’s and our study [22], and suggests that the IPA salt alone may not be the striking element accountable for brood effects. Previously, evidence emerged that adjuvants found in the formulations of plant protection products may not have been inert, as believed, but could be toxic to bees [28]. As these adjuvants are currently regulated differently from active ingredients, it cannot be ruled out that the discrepancy observed here derives from the presence of such substances [50,51].

Thompson et al. [26] further assessed colony conditions with the “Liebefeld method” [34] and obtained results that are in line with those of our field studies. No negative effects on colony growth and overwintering success could be revealed. These findings suggest that glyphosate does not have a lasting effect on honey bee population dynamics but can delay the preadult development of worker bees as a formulated product. Following the method of Schur et al. [31], only one single brood cycle was, however, covered in the mini-hive experiment. Information on the extent to which pupation was delayed by GBH is currently lacking. Considering bee health in a broader view, it appears plausible that all this could lead to an elevated number of viable daughter mites being released from brood cells, boosting *Varroa destructor* infestation. The mite is considered a major threat in apiculture, and this advantage could result in a higher reproductive success [52,53], ultimately affecting bee and colony health. Social buffering plays a substantial part in this context and has been discussed as being a key reason that eusocial insects can withstand stressors such as pesticides and parasites [32,54]. Colony-level functions like honey production, pollination, and overwintering are isolated from the fate of individuals suffering death or other negative impacts by a complex interaction of compensatory mechanisms [23,24,25,55,56]. Simply put, when a colony is exposed to such a stressor, affected bees will die in the worst case. A strong colony will be able to buffer these losses and survive [24]. This is why past laboratory studies most often failed to link effects from a limited number of worker bees—extracted from their hive environment and exposed to pesticide stress—to the fate of an entire colony. Our data highlight that, even though a significant reduction in the body mass of freshly hatched workers was revealed at the high GBH concentration, their survival was not compromised. Furthermore, brood development was altered, but colony growth was maintained. Hence, we urge the need to evaluate at least a second brood cycle and perhaps certain aspects of brood care behavior, including combinatorial approaches of GBH and *Varroa* mites, in order to better understand the underlying mechanisms of the effects observed here.

Retrospectively, there also seems to be a contradictory terminology in the method we followed, one which is used in regulatory risk assessment to evaluate the effects of plant protection products on honey bees [29,30,31]. The term “Brood Termination” implies that development was ended or stopped in certain cells because the expected brood stage was not appropriately achieved (Table S1). A delay, however, is not correctly reflected by this term. Hence, we suggest improving the method and refining its terminology, as new insights for limiting susceptibility to errors have recently emerged [57]. Under semi-field conditions, in particular, high BTRs in control colonies are an issue that is causing high variability within replicates [58]. Suggestions by the ICP-PR Bee Protection Group resulted in improvements of the method; however, sublethal effects were not focused on [59]. With current technical advances, it is possible to enhance the selection process of study colonies, digitize colony and brood assessments, and monitor the flight activity to display sublethal effects more accurately [57,60,61]. This refinement could be highly relevant for the further improvement of regulatory relevant methodologies in order to identify sublethal effects at the colony level and help render the present terminology more precise.

At present, GBH are not subject to many routine assessments of standard bee matrices such as honey and pollen. In this study, we wanted to complement existing residue data that are currently scarce. We revealed that glyphosate residues in pollen are up to 27-fold higher when compared to residues in nectar (collected fresh and from stored in-hive products) deriving from the same application. Our finding is consistent with that of Thompson et al. [26], who presented the same order of magnitude in their greenhouse experiments. Here, glyphosate values were approximately 18-fold higher in pollen when compared to nectar. This deviation cannot be attributed to the chemical properties of glyphosate, the ammonium salt being hydrophilic, and having an amphoteric (dipolar ionic) character [62]. The epicuticular wax on the surface of plant leaves represents a barrier to the polar properties of glyphosate. Hence, so-called surfactants are added to GBH-formulated products to overcome this barrier and increase efficacy [63]. We assume that, due to this fact, pollen shows a many-fold increase in residue levels towards nectar, as the surfactant possibly enriches the active ingredient in the lipophilic matrix of pollen grains. Furthermore, we are in line with the results provided by Thompson et al. [7], Karise et al. [9], and Rubio et al. [10], but our results contradict those of Berg et al. [8], who found many-fold higher glyphosate concentrations in honey than we and the others did. It seems reasonable that, in this particular study [8], honey bees must have been exposed to unusually high levels of GBH, which could be a result of misapplication or misuse of the product. Nevertheless, it became apparent that free-foraging bees were at continuous risk of contamination. In experiment 1, glyphosate residues of 0.18 mg/kg were measured in the pooled control sample from DAT+21. Due to the setup of the mini-hives, drifting of bees was possible, and, even if not obviously visible, robbing could not be ruled out. Notably, in experiment 3, one control and one treatment colony had residues of 0.05 and 0.04 mg glyphosate/kg, respectively, before the application. This indicates that glyphosate was most likely introduced from the environment. However, after seven days, residues were no longer present in the control. From these low numbers, we do not assume side-effects compromising the outcome of our study, but we suggest that one take measures to prevent unintentional contamination in future experiments. A less dense placement of the hives would be a good start to reducing drifting.

Our results further provide a baseline of residues that can be found in a realistic exposure scenario. We point to the fact that glyphosate was persistent for up to 170 days in sugar-based matrices such as honey and syrup. Microbial degradation of glyphosate is prominent for a wide range of gram-positive and negative bacteria, and different classes of fungi (reviewed in [64]). Interestingly, honey has been long-known for its antimicrobial qualities [65]. Its high sugar contents result in low water activity (a_w_), which inhibits or stresses bacterial growth [66]. Due to this characteristic, we suggest that sugar-based matrices are a glyphosate-friendly environment that prevents the degradation of the active ingredient. Moreover, glyphosate is degraded more quickly in phacelia plants grown under field conditions when compared to tunnel plants. This can most probably be attributed to oxidative advanced processes, such as photolysis resulting from ultraviolet (UV) light exposure [67]. The tunnel gauze may have protected the active ingredient from a faster degradation, possibly increasing the action of the herbicide in the tent. This is particularly interesting when considering more harmful substances such as insecticides and should be investigated in greater detail in order to test this hypothesis. Therefore, tunnel exposure very likely represents a worst-case scenario where bees are not only challenged by the confinement but also by the limited and unbalanced forage [30,68]. This outcome can help categorize upcoming findings and provide support for future experiments.

When investigating plant protection products, recent attention has mostly been drawn to neonicotinoid insecticides. Fungicides and herbicides are understudied substance classes when it comes to bee health [69,70], as their mode of action does not generally aim to harm insects. Our findings, however, provide evidence that a formulated herbicide can cause sublethal effects at the colony level. Hence, we think these classes deserve closer attention in future studies. As a lesson learned from past “neonicotinoid research”, we believe a clear focus on field-level data should be emphasized, provided that test systems consider field-realistic exposure scenarios under beekeeping conditions.

## 7. Conclusions

The aim of the present research was to examine the effects of glyphosate-based herbicides at the colony level, with a special focus on worker bee development. Even though we confirmed that GBH did not impact the lifespan of individuals, colony conditions, and overwintering, it became obvious that the herbicide delayed worker brood development when applied at a chronic high concentration. Yet, the mechanisms underlying this surprising effect are not known. However, when considering that stored nectar and beebread are typically utilized for brood rearing, our results need to be interpreted with caution. Bearing in mind the low residues we found in these two matrices, negative effects at the colony level are not likely to be expected when GBH are applied under good agricultural practice. Regardless, we highlight the importance of suitable and relevant approaches to detect the lowest possible effects, which is not necessarily provided with current standard regulatory methods. The refinement of the existing methodology could reveal lasting consequences for honey bee and pollinator health, which we are perhaps not able to observe at present. Further studies are needed to complete the picture of a realistic GBH exposure scenario that challenges not only honey bees but also other pollinators, under field conditions. Ultimately, a pan-European pesticide/hive monitoring could be established, where such results would be merged into a database to serve as information for the public, beekeepers, and decision-makers.

## Figures and Tables

**Table 1:** Glyphosate (GLY) and aminomethylphosphonic acid (AMPA) residues corresponding to the assessment date (DAT = day after treatment) and matrix from all four experiments (T groups only). For the detection limits of the method, see the materials and methods section. All values are rounded and presented as means (±SD) in mg a.i./kg where appropriate (for details of the method, repeat determination, and trace residues found in controls, see Supplementary Materials Method S2 and raw data [46]. *: pooled sample, +: analyzed twice, n.d.: not detectable, -: not measured).

			Stock Solution		Stored Food/Nectar		Honey Sac		Beebread		Corbicular Pollen		Plants	
	Time	Samples (n)	GLY	AMPA	GLY	AMPA	GLY	AMPA	GLY	AMPA	GLY	AMPA	GLY	AMPA
Experiment 1: Brood development & survival	DAT0 (T1)	1	4.764 ± 0.424 ^+^	0.020 ± 0.003 ^+^	-	-	-	-	-	-	-	-	-	-
	DAT0 (T2)	1	137.566 ± 4.318 ^+^	0.428 ± 0.001 ^+^	-	-	-	-	-	-	-	-	-	-
	DAT+21 (T1)	5 *	-	-	5.103	0.013	-	-	-	-	-	-	-	-
	DAT+21 (T2)	4 *	-	-	99.861	0.319	-	-	-	-	-	-	-	-
Experiment 2: Field exposure	DAT0 (+0.5 h)	6 *	-	-	n.d.	n.d.	-	-	-	-	-	-	96.429 ± 13.540 ^+^	0.360 ± 0.005 ^+^
	DAT+1	6	-	-	0.327 ± 0.246	0.087 ± 0.194	-	-	-	-	-	-	44.715 ± 1.429 ^+^	0.255 ± 0.007 ^+^
	DAT+3	6	-	-	0.490 ± 0.455	n.d.	-	-	-	-	-	-	33.634 ± 0.016 ^+^	0.218 ± 0.017 ^+^
	DAT+6	6	-	-	0.347 ± 0.362	n.d.	-	-	5.512 ± 6.922	0.007 ± 0.017	-	-	-	-
	DAT+7	6	-	-	-	-	-	-	-	-	-	-	31.051 ± 1.930 ^+^	0.224 ± 0.007 ^+^
Experiment 3: Overwintering	DAT −1	12	-	-	0.004 ± 0.012	n.d.	-	-	-	-	-	-	-	-
	DAT+6	6	-	-	4.462 ± 1.651	0.006 ± 0.005	-	-	-	-	-	-	-	-
	DAT+13	6	-	-	5.159 ± 1.011	0.012 ± 0.004	-	-	-	-	-	-	-	-
	DAT+43	6	-	-	7.148 ± 3.234	0.033 ± 0.015	-	-	-	-	-	-	-	-
	DAT+170	6	-	-	4.985 ± 0.613	0.019 ± 0.004	-	-	-	-	-	-	-	-
Experiment 4: Residues under semi-field conditions	DAT0 (+1 h)	3 *	-	-	-	-	24.918	0.092	-	-	614.841	2.122	82.407	0.217
	DAT+1	3 *	-	-	-	-	-	-	-	-	-	-	103.892	0.362
	DAT+2	3 *	-	-	-	-	-	-	-	-	-	-	62.845	0.229
	DAT+3	3 *	-	-	-	-	-	-	-	-	-	-	92.886	0.395
	DAT+5	4	-	-	0.182 ± 0.225	n.d.	-	-	-	-	-	-	-	-

## Supplementary Materials

Supplementary materials can be found at https://www.mdpi.com/2075-4450/11/10/664/s1. Table S1: Color code of labeled cell contents (BFD), Figure S1: Pictures of combs with color code, Figure S2: Experimental setup scheme (experiment 1), Figure S3: Selected mean No. of eggs, Figure S4: BTR, Brood Index and Compensation Index from BFD+13 and BFD+21, Figure. S5: Hazard ratio (HR) displayed as a forest plot, Figure S6: Survival probability of untreated mini-hive replicates to justify pooling, Figure S7: Colony conditions Experiment 2, Figure S8: Colony conditions Experiment 3, Method S1: Brood development and photographic assessment adopted from OECD 2007 and based on Schur et al. 2003, Method S2: Analytical method and validation for glyphosate and AMPA.

## Abbreviations

AMPA	Aminomethylphosphonic acid
ANOVA	Analysis of variance
BBCH	Biologische Bundesanstalt für Land—und Forstwirtschaft, Bundessortenamt und CHemische Industrie
BFD	Brood area fixing day
BTR	Brood termination rate
DAT	Day after treatment
EC	European commission
EFSA	European Food Safety Authority
EPPO	European and Mediterranean Plant Protection Organization
GBH	Glyphosate-based herbicides
HPLC	High-performance liquid chromatography
ICP-PR	International Commission for Plant Pollinator Relationships
IPA	Isopropylamine
OECD	Organization for Economic Co-operation and Development
UV	Ultraviolet
