# Supplementary material for: Chronic High Glyphosate Exposure Delays Individual Worker Bee (Apis mellifera L.) Development under Field Conditions
Source: Insects. 2020 Sep 27;11(10):664. doi: 10.3390/insects11100664 (PMC7600025; doi:10.3390/insects11100664)
Supplement: Supplementary file 1 [file insects-11-00664-s001.pdf]

Chronic high glyphosate exposure delays individual worker bee (*Apis mellifera* L.) development under field conditions

Richard ODEMER[1*], Abdulrahim T. ALKASSAB[1], Gabriela BISCHOFF[2], Malte FROMMBERGER[1], Anna WERNECKE[1], Ina P. WIRTZ[1], Jens PISTORIUS[1], Franziska ODEMER[3+]

[1]Julius Kuehn-Institute (JKI), Federal Research Centre for

Cultivated Plants, Institute for Bee Protection, 38104 Brunswick, Germany

[2]Julius Kuehn-Institute (JKI), Federal Research Centre for

Cultivated Plants, Institute for Bee Protection, 14195 Berlin, Germany

[3]Odemer Apiaries, 73765 Neuhausen auf den Fildern, Germany

[+]Present address: Lower Saxony State Office for Consumer Protection and Food Safety, Institute for Apiculture, 29221 Celle, Germany

*Corresponding Author: richard.odemer@julius-kuehn.de

phone: +49 (0)531 299 4218

SUPPLEMENTARY MATERIAL

Tab. S1: Color code of labelled cell contents (BFD)

Fig. S1: Pictures of combs with color code

Fig. S2: Experimental setup scheme (experiment 1)

Fig. S3: Selected mean No. of eggs

Fig. S4: BTR, Brood Index and Compensation Index from BFD+13 and BFD+21

Fig. S5: Hazard ratio (HR) displayed as forest plot

Fig. S6: Survival probability of untreated mini-hive replicates to justify pooling

Fig. S7: Colony conditions Experiment 2

Fig. S8: Colony conditions Experiment 3

Method S1: Brood development and photographic assessment adopted from OECD 2007 and based on Schur et al. 2003

Method S2: Analytical method and validation for glyphosate and AMPA

Supplementary Table S1: Color code of labeled cell contents (BFD)

Tab. S1 Brood area Fixing Day (BFD) assessments during the course of the chronic glyphosate exposure in experiement 1, modified after Schur et al. (2003). The numbers for the brood index were assigned to the cell content, respectively. If the expected brood stage was met at the specified BFD, the cell was labeled "1", if not as terminated (Brood termination = 0). To differentiate brood stages in the photographic assessment, a color code was used (see Fig. S1).

Timing	Expected brood stage	Brood index	Brood termination	Color code
BFD0	egg	1	-	blue
BFD+6*	young (L1-L2) to old larva (L3-L5)	2, 3	if ≠ 2,3	green, red
BFD+13	capped cell (pupa)	4	if ≠ 4	purple
BFD+17*	capped cell (pupa)	4	if ≠ 4	purple
BFD+21	empty cell (e), egg, young larva, nectar (n), pollen (p)	5	if ≠ 1, 2, e, n, p	yellow

Supplementary Figure S1: Pictures of combs with color code

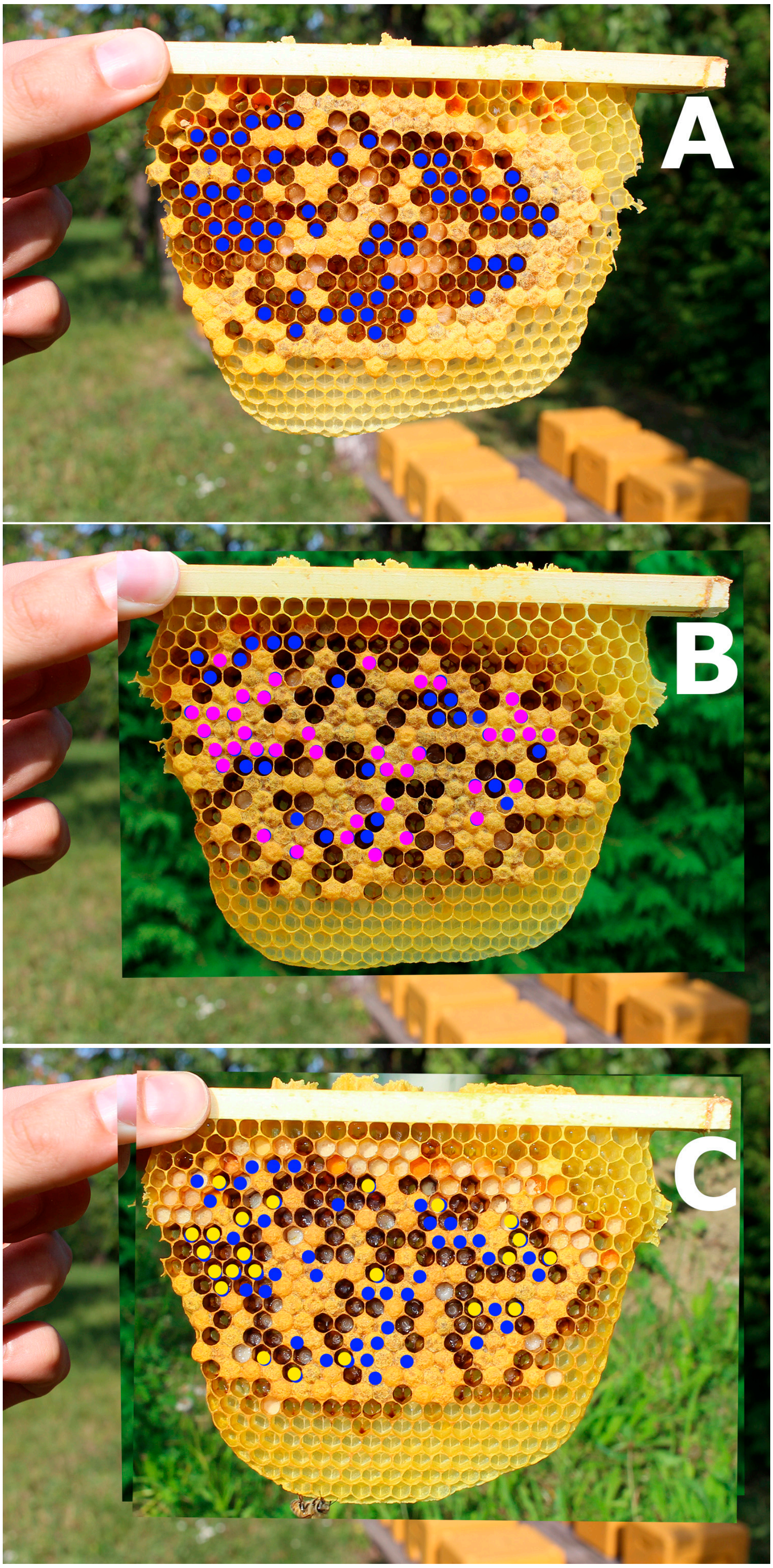

Fig. S1 Pictures from experiment 1, comb no. 4, side A, colony V12, treatment T2. A color code was applied to identify different developmental stages of the brood according to Tab. 1. **A** BFD0, eggs, blue, **B** BFD+13, capped cells (pupae), purple, **C** BFD+21, empty cells OR eggs OR pollen OR nectar OR young larvae, yellow. With this color code, cells could be identified where development was successfully achieved according to Tab. 1. In the case where this was not the case, the development was regarded as terminated. For BFD+13 and BFD+21 the cumulative brood termination rate, brood index and compensation index were calculated for each colony (Fig. S3, Method S1).

Supplementary Figure S2: Experimental setup scheme (experiment 1)

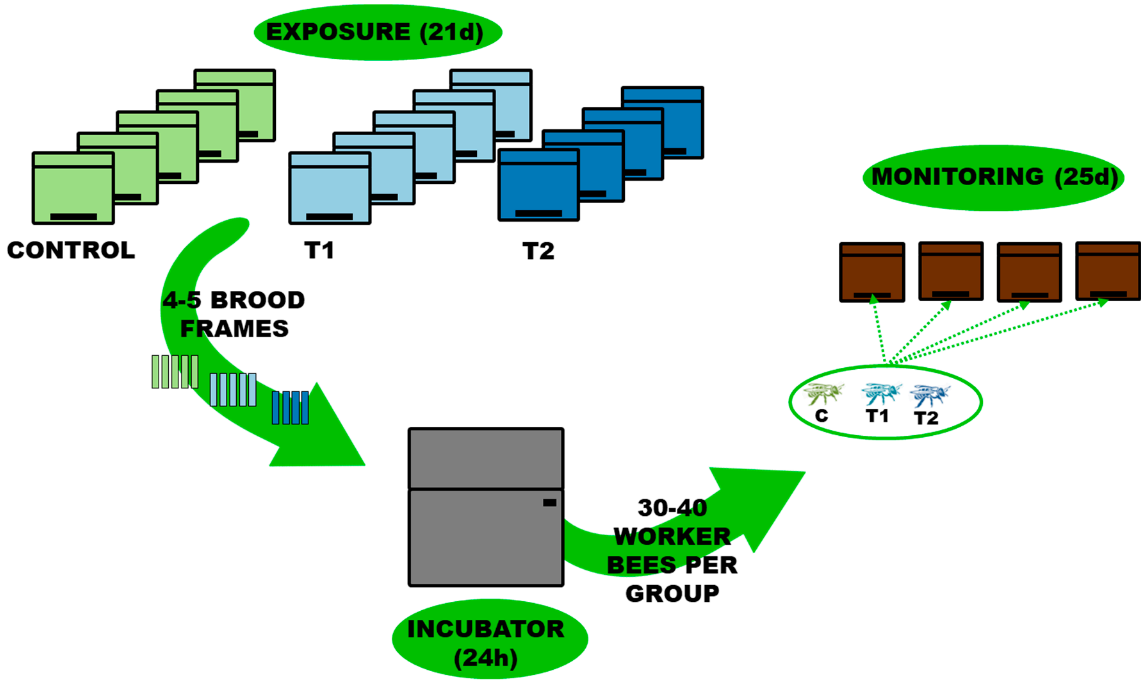

Fig. S2 Experimental setup illustrating the exposure and monitoring period from experiment 1. Control and T1 were comprised of five mini-hives, T2 four. Colonies were exposed to glyphosate for 26 days. Subsequently, one ready-to-hatch brood frame per mini-hive was removed and placed together group-wise in an incubator for 24 h. After hatching, a total of C (n=152), T1 (n=149) and T2 (n=141) bees were marked and subdivided. Approximately 30-40 worker bees per treatment were introduced into four untreated mini-hives, each, and monitored for 25 d.

Supplementary Figure S3: Selected mean No. of eggs

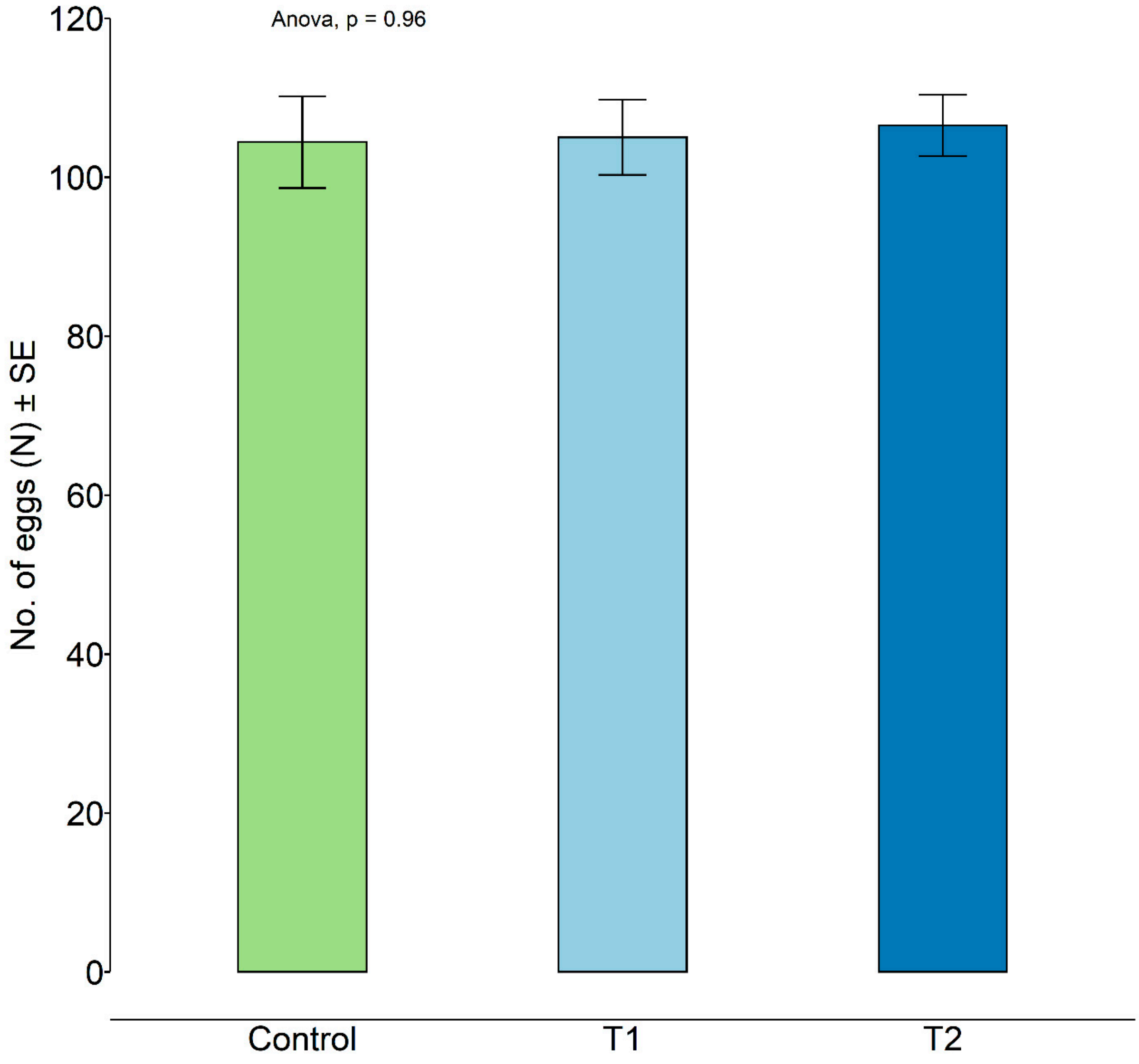

Fig. S3 Barplot displaying mean number of selected eggs in experiment 1for the digital brood assessment on BFD0. All replicates had a similar number of selected eggs (approximately 100; with $P > 0.05$, ANOVA).

Supplementary Figure S4: BTR, Brood Index and Compensation Index from BFD+13 and BFD+21

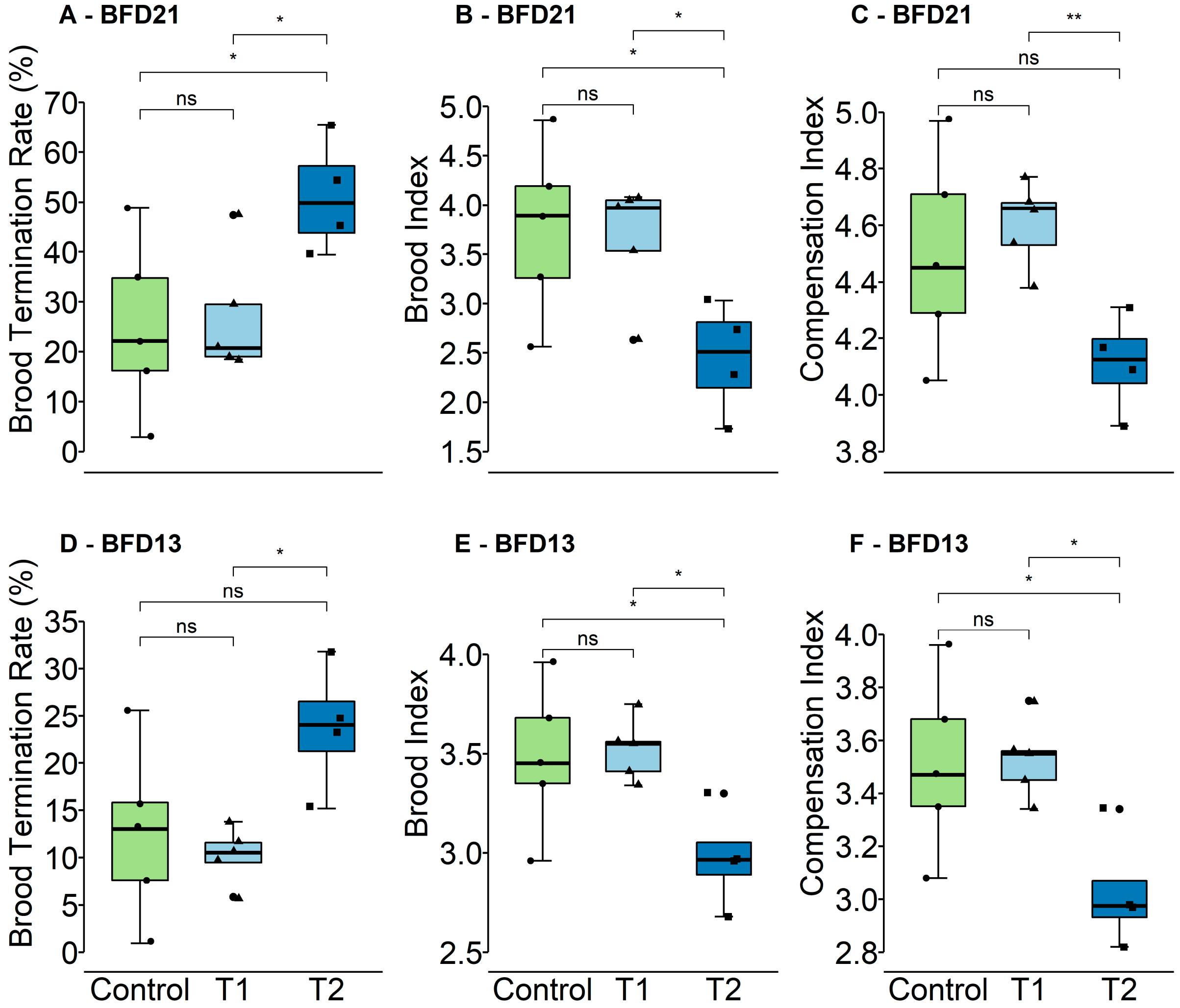

Fig. S4 Here, full details from the brood assessment in experiment 1 are presented modified after Schur et al. (2003) to complement Fig. 3 (see also Method S1). In groups C and T1, successful development was observed in the majority of the marked brood cells. Assuming that at the first assessment only eggs will be marked, the index is 1.0. An increase of the brood index (see paragraph 40) during the following assessment can be observed if a normal development of the brood is presumed. This increase is caused by the development from eggs to larval stages, to the pupae, and finally to the adult, emerged bee, and due to the rising numbers which are assigned to the brood stages (OECD, 2007; with ns = $P > 0.05$ and * = $P < 0.05$, t-test, pairwise).

<u>Supplementary Figure S5:</u> Hazard ratio (HR) displayed as forest plot

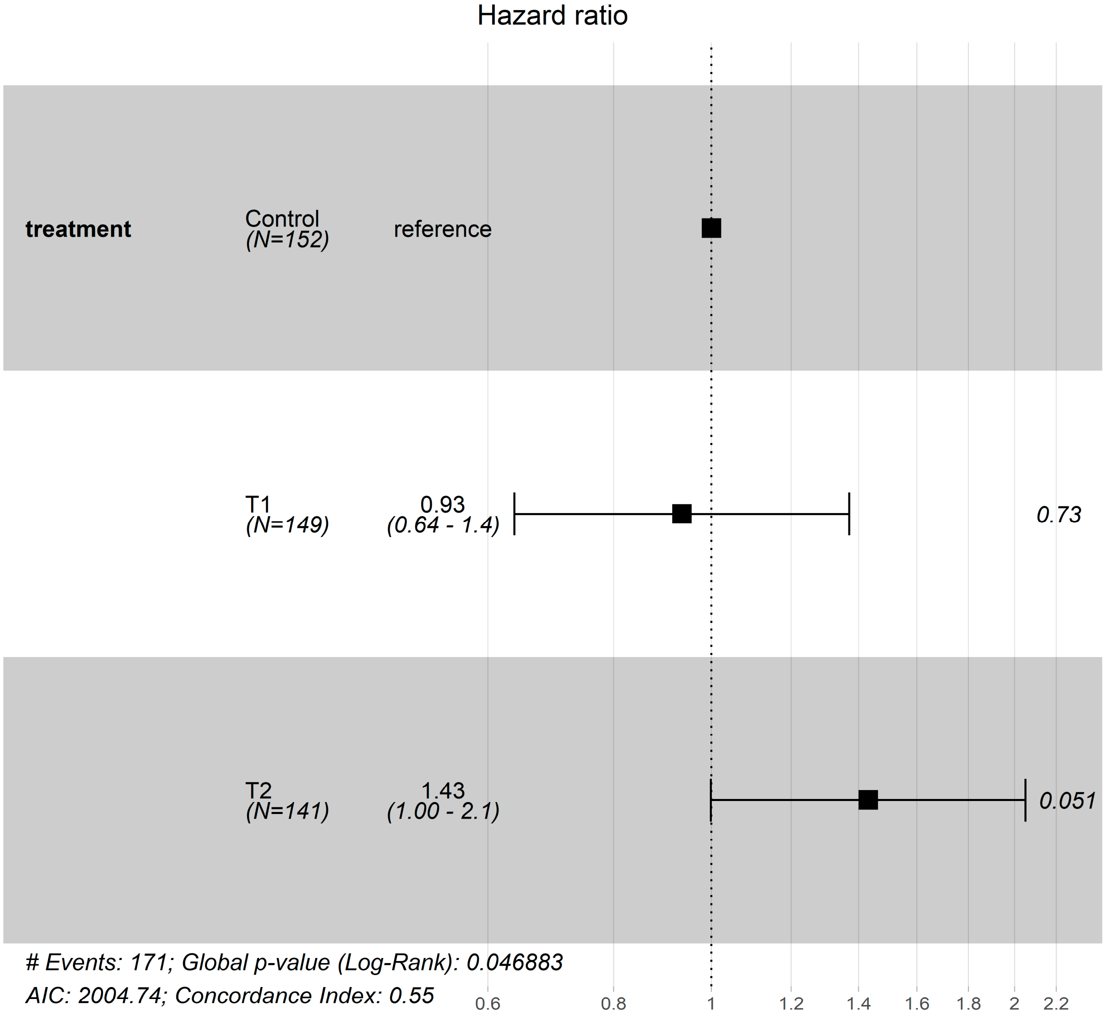

Fig. S5 A Cox proportional hazards model was applied in experiment 1 to determine the hazard ratio (HR) displayed as forest plot. Significant differences within those groups (treatment) were revealed close to the statistical threshold (global *P* = 0.047, log-rank test). A pairwise comparison, however, did not confirm these differences between the respective groups. With an HR of 0.93 for T1 and 1.43 for T2, the treated bees were not at risk of dying sooner when compared to the control (T1-C: P = 0.73, T2-C: *P* = 0.051, Log-rank test)

<u>Supplementary Figure S6:</u> Survival probability of untreated mini-hive replicates to justify pooling

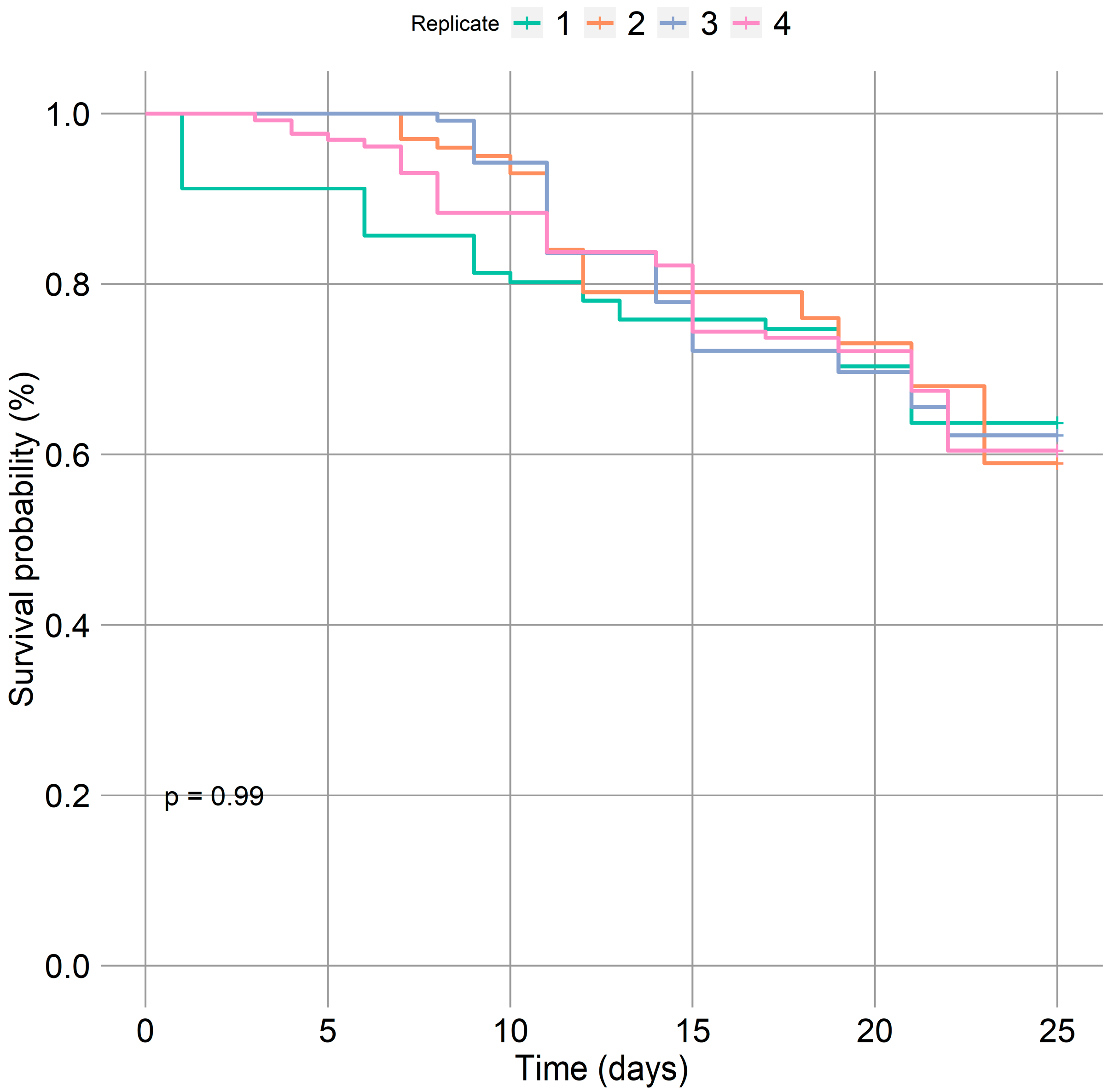

Fig. S6 To justify pooling bees from the same groups but different mini-hives for survival analysis in experiment 1, these hives were evaluated separately treated as replicates. The test showed no significant differences ($P > 0.05$, Log-rank test).

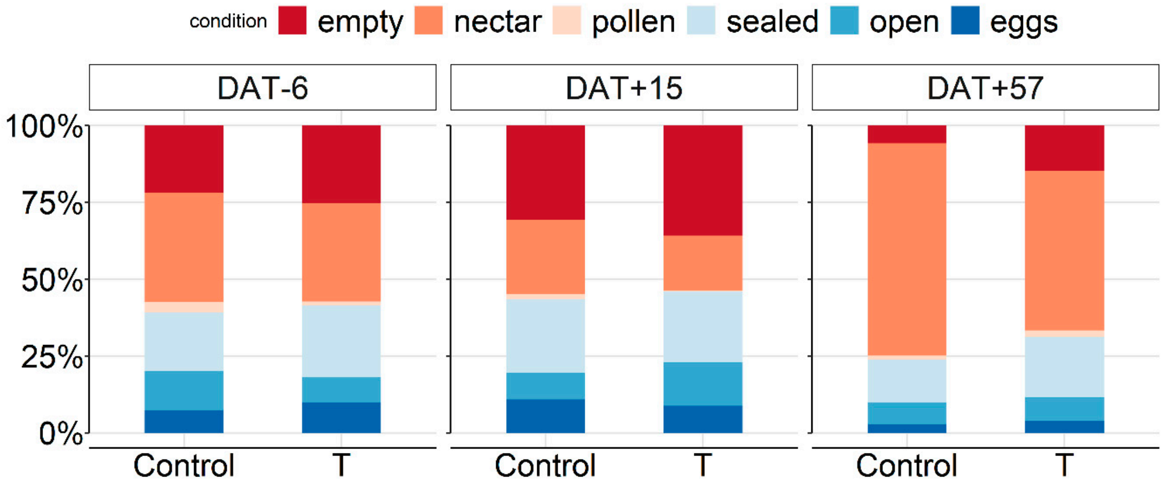

Fig. S7 Colony conditions experiment 2: Proportion of empty cells, stores (nectar, pollen), and worker brood cells (eggs, open, sealed) presented as total colony condition of both groups (control C, glyphosate treatment T) corresponding to their assessment date (DAT = day after treatment).

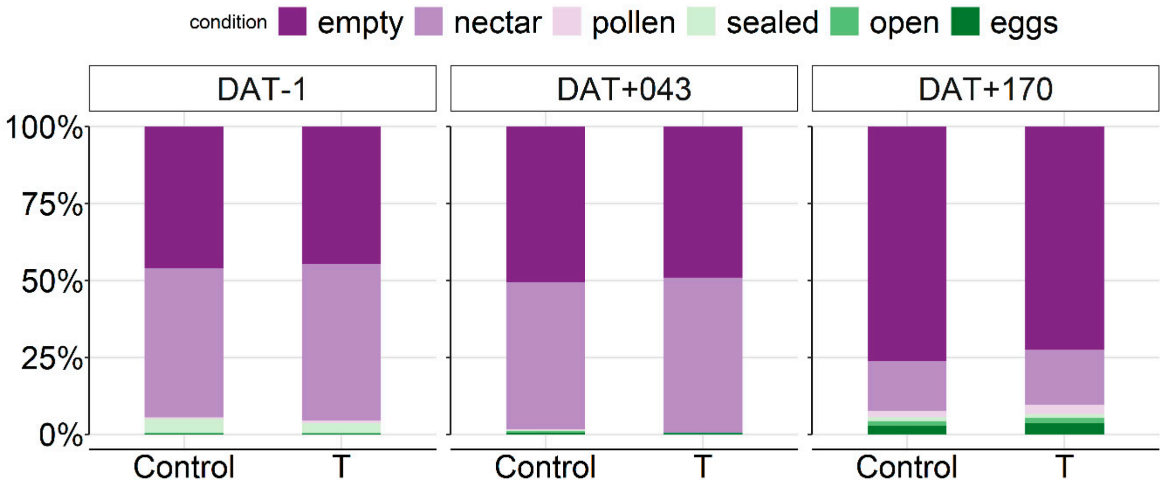

Fig. S8 Colony conditions experiment 3: Proportion of empty cells, stores (nectar, pollen), and worker brood cells (eggs, open, sealed) presented as total colony condition of both groups (control C, glyphosate treatment T) corresponding to their assessment date (DAT = day after treatment).

<u>Supplementary Method M S1:</u> Brood development and photographic assessment adopted from OECD 2007 and based on Schur et al. 2003

Brood termination rate

Based on the brood termination-rate the failure of individual eggs or larvae to develop is quantitatively assessed. For the calculation of the brood termination rate the observed cells are split into two categories:

- The bee brood in the observed cell reached the expected brood stage at the different assessment days or was found empty or containing an egg after hatch of the adult bee on BFD +22 → successful development
- The bee brood in the observed cell did not reach the expected brood stage at one of the assessment days or food was stored in the cell during BFD +5 to +16 → termination of the bee brood development

For the final calculation the number of cells, where termination of the bee brood development was recorded, is summed up for each treatment and colony, is multiplied by 100 and divided by the number of cells observed to obtain the brood termination rate in %.

Brood index

The brood index is an indicator of the bee brood development and facilitates comparison between different treatments. The brood-index is calculated for each assessment day and colony. Therefore, the brood development in each cell will be checked starting from BFD 0 up to BFD +22. The cells are classified from 1 to 5 as described in paragraph 33 (Tab. S1, OECD 2007) if the cells contain the expected brood stage at the different assessment days. If a cell does not contain the expected brood stage or food is stored in the cell during BFD +5 to +16 (see Table 4, OECD 2007) the cell has to be counted 0 (see Table 5, OECD 2007) at that assessment day and also on the following days, irrespective whether the cell is filled again with brood. This might require a further transformation of a value as described in paragraph 33. For the final calculation the values of all individual cells in each treatment, assessed on the same day, are summed up and divided by the number of observed cells to obtain the average brood index.

Compensation index

The compensation index is an indicator of the recovery of the colony and will also be calculated for each assessment day and colony. The cells are classified from 1 to 5 as described in paragraph 33 (Tab. S1, OECD 2007), solely based on the identified growth stage on the assessment days. By that, the compensation of bee brood losses will be included in the calculation of the indices. For the final calculation the values of all individual cells in each treatment, assessed on the same day, are summed up and divided by the number of observed cells to obtain the average compensation index.

<u>Supplementary Method M S2:</u> Analytical method and validation for glyphosate and AMPA

Glyphosate analysis

Preparation of feeding solution

A sample of 500 mg (approx. 400 µl) was weighed in a plastic tube (15 ml) and 9600 µl of the extracting agent (50 mM acetic acid/10 mM Na_2EDTA) were added. The tubes were closed and shaken thoroughly. Depending on the active substance concentration in the feeding solutions, these solutions were measured undiluted (control samples) or diluted to different extents. The dilutions were made with the extracting agent while adding the internal standards.

Preparation of honey samples

A sample of approx. **1 g** was weighed in a plastic tube (15 ml) and a surrogate standard solution (20 µl Glufosinate (conc.: 2.5 ng/µl, corresponding to 10 pg/µl in the measuring solution)) and **4.3 ml** of the extracting agent (50 mM acetic acid/10 mM Na_2EDTA) were added to the sample. The tubes were closed and after homogenization using a Vortex-mixer left to stand for 30 minutes. Afterwards the tubes were shaken for one minute by hand, further 10 minutes with a horizontal shaker, and then centrifuged for 5 minutes (1690 g). The entire supernatant was removed and filtered respectively cleaned using a Solid Phase Extraction (SPE) cartridge (OASIS HLB 6cc, 200 mg; Waters), to retain parts of the sugar in the sample. Before use, the SPE cartridge was conditioned with 2 ml methanol and 2 ml extracting agent and let run dry to not dilute the sample. 1000 µl of the sample extract were filled into a vial for measuring and 20 µl of an internal standard solution (glyphosate$^{13}C_2{}^{15}N$, AMPA$^{13}C^{15}N$, conc.: 1 ng/µl each, corresponding to 20 pg/µl in the measuring solution) were added. The measurements were started immediately after the completion of the extracts.

Preparation of pollen samples

The samples were analyzed in the same way as described for honey with only one difference. **5.0 ml** of the extracting agent (50 mM acetic acid/10 mM Na_2EDTA) were added to the sample.

Preparation of plant samples

A sample of **10 g** was weighed in a plastic cup (100 ml) and a surrogate standard solution (100 µL Glufosinate (conc.: 5 ng/µl, corresponding to 10 pg/µl in the measuring solution) and **50 ml** of the extracting agent (50 mM acetic acid/10 mM Na_2EDTA) were added. The cups were closed, left to stand for 30 minutes and then shaken for 20 minutes using a horizontal shaker.

Subsequently, the plant samples were crushed using a disperser and simultaneously extracted for 3 minutes. Then the samples were filtered using folded filters (particle retention 5 - 8 µm) into a 50 ml tube, or alternatively, the plant extracts were centrifuged (10 min, 1.690 g). **5 ml** of the filtered respectively centrifuged samples were transferred onto a conditioned (see the preparation of honey samples) SPE-cartridge and filtered again respectively further cleaned. 1000 µl of the extract were prepared for measurements as described above (preparation of honey samples).

Identification and quantification of the residues in the samples

LC-MS/MS was used for the identification and quantification of the target substances in the samples. Three multiple reaction monitoring (MRM) transitions were monitored for each analyte in order to confirm compound identity.

Reference standards in matrix and/or extracting agent were used for quantification, which was carried out according to the method of the internal standard. A large number of standards with the following concentrations were measured to create the calibration function: 0.1, 0.2, 0.5, 1, 5, 10, 25, 50, 100, 200, 500 and 1000 pg/µl). The results shown for the samples are averages of duplicate injections of sample extracts.

In undiluted and 1:10 diluted samples, the analyte contents were determined using matrix-matched standards. If samples had to be diluted 1:100 or 1:1000, the analytes were quantified using reference standards in extracting agent as matrix effects were sufficiently reduced by dilution.

The results for the surrogate standard glufosinate were used to control the analysis and were not included in the calculation of the analyte content in the samples.

Equipment and measurement conditions

LC-MS/MS

The system used was a Nexera X2 HPLC system (SHIMADZU Corp., Kyoto, Japan) coupled to a triple quadrupole mass spectrometer Q TRAP 6500+ (SCIEX, Framingham, MA, USA) equipped with an electrospray ionization (ESI) source.

The mass spectrometric parameters were as follows:

- Scan type: sMRM
- Polarity: negative
- Ion spray voltage: - 4500 V
- Source temperature: 700 °C
- Curtain gas: 30 psi
- Nebulizer Gas (GS 1): 60 psi
- Heater Gas (GS 2): 60 psi
- Collision gas (nitrogen): high

The chromatographic separations were performed on an Acclaim Trinity Q1 column (3.0 x 100 mm; 3 µm, Thermo Fisher Scientific) with a pre-column SecurityGuard C_{18} (3.0 x 4 mm, Phenomenex). The column oven temperature was set to 35 °C and the autosampler tray temperature was set to 15°C.

First, the samples were analyzed with the mobile phases (A) acetonitrile and (B) ultrapure water (0,055 µS/cm) with 50 mM ammonium formiate (adjusted to pH=2.9 with formic acid). The injection volume was 10 µl. The flow rates and gradient I and are shown in Tab. MS1.

Later the chromatographic conditions were optimized (Chamkasem & Vargo, 2017) and the samples analyzed with the mobile phases (A) ultrapure water and (B) ultrapure water with 50 mM ammonium formiate (adjusted to pH=2.9 with formic acid). A diverter valve between the LC column and the MS interface was used to direct the LC eluent to waste just before the AMPA peak (1.9 min) and after the glyphosate peak (3.5 min). The injection volume was 20 µl. The flow rates and gradient II are shown in Tab. MS2.

MRM transitions (negative mode) and compound dependent parameters are shown in Tab. MS3.

Tab. MS1: Gradient I

Time (min)	Flow (ml/min)	Mobile phase A (%)	Mobile phase B (%)
0.01	0.5	0	100
3.00	0.5	0	100
3.20	0.5	100	0
6.00	0.5	100	0
6.20	0.5	0	100
10.20	0.5	0	100

Tab. MS2: Gradient II

Time (min)	Flow (ml/min)	Mobile phase A (%)	Mobile phase B (%)
0.01	0.5	100	0
0.50	0.5	100	0
0.51	0.5	0	100
4.00	0.5	0	100
4.10	0.7	100	0
10.00	0.7	100	0

Tab. MS3: MRM-transitions (negative mode) and compound dependent parameters.

Compound	Precursor Ion Q1 Mass (m/z)	Product Ion Q3 Mass (m/z)	DP (V)	CE (V)	CXP (V)
Glyphosate 1	168	63	-30	-26	-7
Glyphosate 2	168	150	-30	-14	-9
Glyphosate 3	168	81	-30	-20	-9
AMPA 1	110	63	-15	-24	-7
AMPA 2	110	81	-15	-18	-9
AMPA 3	110	79	-15	-34	-9
Glufosinate 1 (Surr)	180	63	-50	-66	-13
Glufosinate 2	180	95	-50	-24	-13
Glufosinate 3	180	85	-50	-24	-15

Compound	Precursor Ion Q1 Mass (m/z)	Product Ion Q3 Mass (m/z)	DP (V)	CE (V)	CXP (V)
Glyphosate-$^{13}C_2{}^{15}N$ 1	171	63	-50	-26	-7
Glyphosate-$^{13}C_2{}^{15}N$ 2	171	153	-50	-14	-9
AMPA$^{13}C^{15}N$ 1	112	63	-45	-24	-7
AMPA$^{13}C^{15}N$ 3	112	79	-45	-38	-9

DP =Declustering Potential, CE = Collision Energy, CXP = collision cell exit potential, Surr = surrogate standard

Method validation - glyphosate

The validation study was performed to evaluate recoveries (REC), detection limits (LOD) and quantification limits (LOQ). Control samples of honey (used for honey stomach as well), pollen and plant material (phacelia) were fortified at different levels and 5 (4 respectively 7 in the case of plants) replicates extracted as described above. For the determination of recovery rates, detection and quantification limits, reference standards in solvent and matrix were prepared with the following concentration levels: 0.1, 0.25, 0.5, 1, 2.5, 5, 10, 25, 50, 100, 250 and 500 pg/µl.

The LOD was determined as the lowest concentration at which at least two MRM were detected, the peak signals of which were three times higher than the background noise of the chromatogram and the ratio of which was in the range of the required criteria (SANTE, 2019). The next highest concentration of the calibration standards above the detection limit was set as LOQ.

The results of the method validation procedure with honey, pollen and plant material are summarized in the following tables.

Tab. MS4: REC, RSD, LOD and LOQ of the target analytes in honey

**Honey**	**Fortification level**									
	**50 µg/g** [1] (n=5)		**250 µg/kg** (n=5)		**25 µg/kg** (n=5)		Standards in extracting agent		Standards in honey matrix	
**Analytes**	REC [%]	RSD [%]	REC [%]	RSD [%]	REC [%]	RSD [%]	LOD [1] [µg/kg]	LOQ [1] [µg/kg]	LOD [µg/kg]	LOQ [µg/kg]
Glyphosate	112	17	85	11	74	13	5.0	10	5.0	12.5
AMPA	110	14	85	9	77	10	2.5	5.0	2.5	5.0

[1] At a honey weight of 1 g and a 1:1000 dilution of the sample.

Table MS5: REC, RSD, LOD and LOQ of the target analytes in pollen

Pollen	Fortification level									
	**250 µg/kg** (n=5)		**50 µg/kg** (n=5)		**25 µg/kg** (n=5)		Standards in extracting agent		Standards in pollen matrix	
**Analytes**	REC [%]	RSD [%]	REC [%]	RSD [%]	REC [%]	RSD [%]	LOD [µg/kg]	LOQ [µg/kg]	LOD [µg/kg]	LOQ [µg/kg]
Glyphosate	79	13	80	4	148	18	5.0	10	12.5	25
AMPA	71	16	74	7	87	13	2.5	5.0	12.5	25

Table MS6: REC, RSD, LOD and LOQ of the target analytes in plant material

Plants (Phacelia)	Fortification level									
	**250 µg/kg** (n=4)		**50 µg/kg** (n=7)		**25 µg/kg** (n=4)		Standards in extracting agent		Standards in phacelia matrix	
**Analytes**	REC [%]	RSD [%]	REC [%]	RSD [%]	REC [%]	RSD [%]	LOD [µg/kg]	LOQ [µg/kg]	LOD [µg/kg]	LOQ [µg/kg]
Glyphosate	83	6	74	8	73	10	5.0	10	5.0	12.5
AMPA	83	5	77	8	82	9	2.5	5.0	12.5	25

References

OECD (Organisation for Economic Co-operation and Development) Guidance Document on the honey bee (*Apis mellifera L.*) brood test under semi-field conditions No. 75. *ENV/JM/MONO* **2007**, *OECD Envir*.

Schur, A.; Tornier, I.; Brasse, D.; Mühlen, W.; Von Werner Ohe, D.E.R.; Wallner, K.; Wehling, M. Honey bee brood ring-test in 2002: Method for the assessment of side effects of plant protection products on the honey bee brood under semi-field conditions. *Bull. Insectology* **2003**, *56*, 91–96.

SANTE/12682/2019 Method Validation and Quality Control Procedures for Pesticide Residues Analysis in Food and Feed. *EU Ref. Lab. Residues Pestic.* **2020**, *Implemente*. https://www.eurl-pesticides.eu/userfiles/file/EurlALL/AqcGuidance_SANTE_2019_12682.pdf

Chamkasem, N.; Vargo, J.D. Development and Independent Laboratory Validation of an Analytical Method for the Direct Determination of Glyphosate, Glufosinate, and Aminomethylphosphonic Acid in Honey by Liquid Chromatography/Tandem Mass. Spectrometry. *J. Regul. Sci.* **2017**, *5*, 5 (2) 1-9.
